# A B-box protein BReaks brassinosteroid regulation of photomorphogenesis in the dark and light

**DOI:** 10.1093/plphys/kiag593

**Published:** 2026-08-12

**Authors:** Gunjan Sharma, Akanksha Bhatnagar

**Affiliations:** Assistant Features Editor, Plant Physiology, American Society of Plant Biologists, Rockville, United States; School of Biosciences, University of Birmingham, Edgbaston B15 2TT, United Kingdom; School of Biosciences, University of Birmingham, Edgbaston B15 2TT, United Kingdom

In nature, seeds are buried in the soil and must be prepared to emerge into the light. Light triggers a developmental switch from dark-dependent growth (skotomorphogenesis) to light-dependent growth (photomorphogenesis). Different photoreceptors such as phytochromes, cryptochromes, phototropins, and UV RESISTANCE LOCUS 8 perceive light and relay signals to their downstream players (Franklin and Quail 2010). Elongated hypocotyl 5 (HY5) is a key transcription factor (TF) promoting photomorphogenesis by regulating the expression of several target genes and itself (Gangappa and Botto 2016; Bhatnagar et al 2020). In Arabidopsis, the 32-member *BBX* TF family is divided into subgroups based on domain architecture and phylogenetic relationships. BBX TFs regulate a wide range of physiological and developmental processes, including photomorphogenesis (Khanna et al 2009).

Brassinosteroids (BRs), among other hormones, promote skotomorphogenesis (Nolan et al 2020). However, how light and BR signaling pathways regulate early seedling development in Arabidopsis is not fully understood. In this issue of *Plant Physiology*, Rahul et al (2026) addressed this knowledge gap and showed an interplay between BBX13 and BR to promote photomorphogenesis for efficient photosynthesis during the dark-to-light transition. *proBBX13:GUS* is expressed predominantly in cotyledons of 2- to 4-d-old dark-grown seedlings and then declines after day 5, suggesting a possible role in skotomorphogenesis. To functionally characterize a role for BBX13 in skotomorphogenesis, the authors employed overexpression/mutant lines and observed cotyledon opening and hypocotyl elongation as parameters of skoto- and photomorphogenesis. Overexpression of *BBX13* increased cotyledon opening and reduced hypocotyl elongation in 7-d-old dark-grown seedlings on sucrose-deficient media as compared to wild-type (WT) and *bbx13* mutants. Interestingly, addition of sucrose reversed the phenotype, and *BBX13-OE* showed a closed cotyledon phenotype. Sucrose triggers accumulation of BR in the dark, while light inhibits this accumulation (Zhang et al 2016; Zhang et al 2021). Together, these observations suggest that *BBX13* negatively regulates skotomorphogenesis, likely by affecting BR signaling.

To assess the antagonistic role of BBX13 with BR, the authors observed the cotyledon opening phenotype of *BBX13* overexpression/mutant lines in brassinazole-supplemented media, a chemical inhibitor of BR synthesis. BR is known to inhibit cotyledon opening, and brassinazole treatment opens cotyledons. The *bbx13* mutants exhibited hyposensitivity, displaying reduced opening of cotyledons, while overexpressors exhibited hypersensitivity with greater cotyledon opening, suggesting that *BBX13* dampens the BR-mediated dark growth phenotype of cotyledon closure.

BRASSINAZOLE RESISTANT 1 (BZR1) is a positive regulator of BR signaling that promotes skotomorphogenesis in the dark (Nolan et al 2020). The authors showed that BBX13 physically interacted with BZR1, and further genetic characterization of *brassinazole-resistant 1-1D* (*bzr1-1D*) mutants with constitutive overexpression of *BZR1* showed reduced cotyledon opening. Thus, high levels of BZR1 can reverse the cotyledon opening conferred by *BBX13-OX*. Furthermore, BZR1 negatively regulates *BBX13* transcript levels, implicating a negative feedback loop. In the dark, BBX13 blocks BZR1 from inhibiting cotyledon opening, and BZR1 counters by repressing *BBX13* gene expression.

The role of BBX13 in light-dependent growth needed further investigation. *BBX13* transcript levels decreased after 3 h of light exposure during a dark-to-light transition. Despite the decreased expression of the native *BBX13* gene, *BBX13-OE* lines demonstrated a phenotype in the light. The cotyledon opening angle of *BBX13-OE* was wider than WT, while the mutants displayed an opposite phenotype under all light conditions. Additionally, expression of target genes involved in cotyledon opening, such as *SAUR 14*, *16*, and *50*, was significantly induced in *BBX13-OE* lines.

Since chlorophyll biosynthesis and shorter hypocotyl length are critical morphological aspects of the dark-to-light transition, the authors quantified dark-accumulated chlorophyll precursor protochlorophyllide and light-induced hypocotyl shortening. *BBX13* overexpression lines accumulated the highest protochlorophyllide content and exhibited shorter hypocotyls, followed by the WT and *bbx13* mutants, suggesting a positive role of *BBX13* in dark-induced protochlorophyllide accumulation and light-induced hypocotyl shortening. These observations suggest that BBX13 regulates the optimal protochlorophyllide level, facilitating chlorophyll biosynthesis without photosensitivity.

HY5, along with other BBX proteins, promotes light-dependent seedling growth (Xu 2020). Surprisingly, BBX13 did not physically interact with HY5 in the yeast two-hybrid system. However, HY5 bound to a G-box motif in the *BBX13* promoter, potentially upregulating its expression, as *BBX13* expression was reduced in *hy5*. Further observations of hypocotyl length and cotyledon opening angle of *hy5bbx13* double mutants showed a longer hypocotyl length and reduced cotyledon opening angle, suggesting genetic additivity. Moreover, *BBX13-OE* in *hy5* mutants rescued the *hy5* phenotype, suggesting that BBX13 regulates photomorphogenesis by inhibiting hypocotyl length and promoting cotyledon opening downstream of HY5.

In conclusion, BBX13 coordinates the transition from skotomorphogenesis to photomorphogenesis by regulating antagonistic interplay between light and BR signaling pathways. In the dark, BBX13 antagonizes BR-mediated skotomorphogenic development by promoting cotyledon opening, chlorophyll precursor protochlorophyllide accumulation, and hypocotyl shortening via its interaction with BZR1. However, in the light, BBX13 promotes photomorphogenesis by inhibiting hypocotyl elongation and promoting cotyledon opening and greening in a HY5-dependent manner (Fig. 1). The authors have increased our understanding of the optimization of plant morphology during soil emergence through BBX13 and BR interplay. Further investigations at the tissue and cellular levels would underpin the mechanisms used by BBX13 to influence different developmental programs.

**Figure 1:**
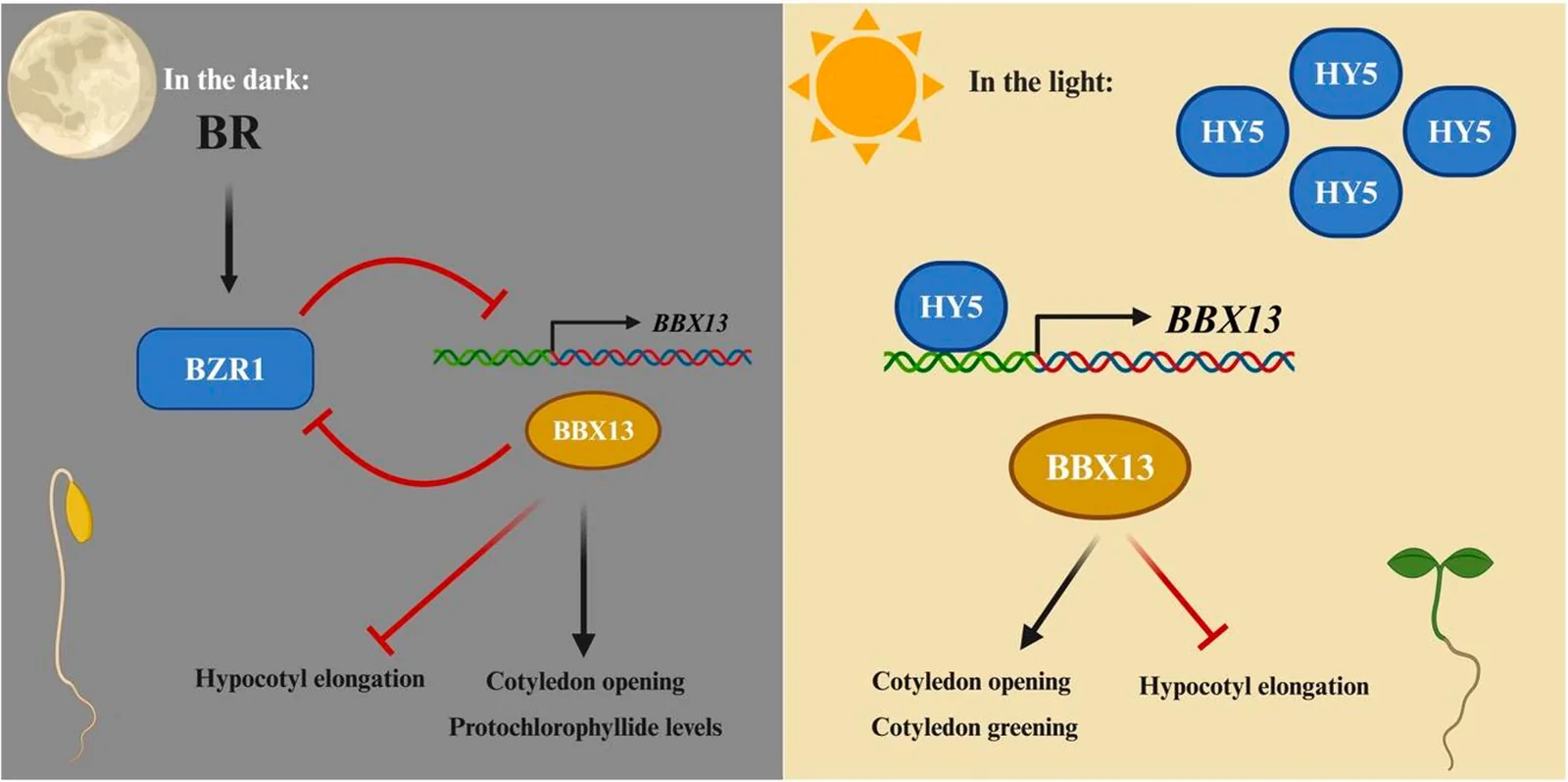
Proposed model of BBX13 in regulating photomorphogenesis in Arabidopsis under dark and light conditions. During dark, BBX13 regulates hypocotyl elongation and a closed cotyledon phenotype by inhibiting BZR1-mediated BR signaling through a negative feedback loop. However, during light, HY5 induces the expression of *BBX13* via direct binding to the *G-box* elements, leading to light-induced growth by activating cotyledon opening, greening, and hypocotyl shortening. Adapted from Rahul et al (2026).

Related articles published in *Plant Physiology:*

Zhao et al (2024) have uncovered a dual function of HOMEODOMAIN ARABIDOPSIS 1 protein in regulating BR biosynthesis and auxin signaling critical for promoting dark- and light-dependent growth and development.

Mukherjee et al (2026) have demonstrated a link between light-controlled developmental programs with stress-responsive pathways for plant stress resilience during dynamic environmental fluctuations.

## Data Availability

No new data were generated or analyzed for this article.
